# Progress Toward Regional Measles Elimination — Worldwide, 2000–2019

**DOI:** 10.15585/mmwr.mm6945a6

**Published:** 2020-11-13

**Authors:** Minal K. Patel, James L. Goodson, James P. Alexander, Katrina Kretsinger, Samir V. Sodha, Claudia Steulet, Marta Gacic-Dobo, Paul A. Rota, Jeffrey McFarland, Lisa Menning, Mick N. Mulders, Natasha S. Crowcroft

**Affiliations:** ^1^Department of Immunization, Vaccines, and Biologicals, World Health Organization, Geneva, Switzerland; ^2^Global Immunization Division, Center for Global Health, CDC; ^3^Division of Viral Diseases, National Center for Immunization and Respiratory Diseases, CDC.

In 2010, the World Health Assembly (WHA) set the following three milestones for measles control to be achieved by 2015: 1) increase routine coverage with the first dose of measles-containing vaccine (MCV1) among children aged 1 year to ≥90% at the national level and to ≥80% in every district, 2) reduce global annual measles incidence to <5 cases per 1 million population, and 3) reduce global measles mortality by 95% from the 2000 estimate* (*1*). In 2012, WHA endorsed the Global Vaccine Action Plan,^†^ with the objective of eliminating measles^§^ in five of the six World Health Organization (WHO) regions by 2020. This report describes progress toward WHA milestones and regional measles elimination during 2000–2019 and updates a previous report (*2*). During 2000–2010, estimated MCV1 coverage increased globally from 72% to 84% but has since plateaued at 84%–85%. All countries conducted measles surveillance; however, approximately half did not achieve the sensitivity indicator target of two or more discarded measles and rubella cases per 100,000 population. Annual reported measles incidence decreased 88%, from 145 to 18 cases per 1 million population during 2000–2016; the lowest incidence occurred in 2016, but by 2019 incidence had risen to 120 cases per 1 million population. During 2000–2019, the annual number of estimated measles deaths decreased 62%, from 539,000 to 207,500; an estimated 25.5 million measles deaths were averted. To drive progress toward the regional measles elimination targets, additional strategies are needed to help countries reach all children with 2 doses of measles-containing vaccine, identify and close immunity gaps, and improve surveillance.

## Immunization Activities

WHO and the United Nations Children’s Fund (UNICEF) determine vaccination coverage using data from administrative records (calculated by dividing the number of vaccine doses administered by the estimated target population, reported annually) and vaccination coverage surveys, to estimate MCV1 and second dose measles-containing vaccine (MCV2) coverage through routine (i.e., not through mass campaigns) immunization services.^¶^ During 2000–2010, estimated MCV1 coverage increased worldwide from 72% to 84%; however, coverage has remained at 84%–85% since 2010, with considerable regional variation (Table 1).

**TABLE 1:** Estimates of coverage with the first and second dose of measles-containing vaccine administered through routine immunization services, reported measles cases, and incidence by World Health Organization (WHO) region — worldwide, 2000, 2010, 2016, and 2019

WHO region/Year (no. of countries in region)	Percentage				No. of reported measles cases^†^	Measles incidence per 1 million population^†,§^
	MCV1* coverage	Countries with ≥90% MCV1 coverage	MCV2* coverage	Reporting countries with <5 measles cases per 1 million population		
**African**						
2000 (46)	53	9	5	8	520,102	836
2010 (46)	73	37	4	30	199,174	232
2016 (47)	69	34	23	51	36,269	37
2019 (47)	69	32	33	34	618,595	567
**Americas**						
2000 (35)	93	63	65	89	1,754	2
2010 (35)	93	74	67	100	247	0.3
2016 (35)	92	66	80	100	97	0.1
2019 (35)	88	71	75	91	19,244	28
**Eastern Mediterranean**						
2000 (21)	71	57	28	17	38,592	90
2010 (21)	77	62	52	40	10,072	17
2016 (21)	82	57	74	55	6,275	10
2019 (21)	82	52	75	42	18,458	27
**European**						
2000 (52)	91	62	48	45	37,421	50
2010 (53)	93	83	80	69	30,625	34
2016 (53)	93	81	88	82	4,440	5
2019 (53)	96	85	91	32	105,755	116
**South-East Asia**						
2000 (10)	63	30	3	0	78,558	51
2010 (11)	83	45	15	36	54,228	30
2016 (11)	89	64	75	27	27,530	14
2019 (11)	94	73	83	30	29,239	15
**Western Pacific**						
2000 (27)	85	48	2	30	177,052	105
2010 (27)	96	63	87	68	49,460	27
2016 (27)	96	63	91	68	57,879	31
2019 (27)	94	67	91	46	78,479	41
**Totals**						
**2000 (191)**	**72**	**45**	**18**	**38**	**853,479**	**145**
**2010 (193)**	**84**	**63**	**42**	**60**	**343,806**	**50**
**2016 (194)**	**85**	**61**	**67**	**70**	**132,490**	**18**
**2019 (194)**	**85**	**63**	**71**	**46**	**869,770**	**120**

**Abbreviations:** MCV1 = routine first dose of measles-containing vaccine; MCV2 = routine second dose of measles-containing vaccine.

* http://www.who.int/immunization/monitoring_surveillance/data/en; data as of July 15, 2020.

^†^ http://apps.who.int/immunization_monitoring/globalsummary/timeseries/tsincidencemeasles.html; data as of July 15, 2020.

^§ ^Population data from United Nations, Department of Economic and Social Affairs, Population Division, 2020. Any country not reporting data on measles cases for that year was removed from both the numerator and denominator in calculating incidence.

Among 194 WHO member states, 122 (63% of member states) achieved ≥90% MCV1 coverage in 2019, a 42% increase from 86 (45%) countries in 2000, but a 4% decrease from a peak of 127 (65%) countries in 2012. In 2019, 42 (22%) countries achieved MCV1 coverage ≥90% nationally and ≥80% in all districts**; however, during that year 19.8 million infants did not receive MCV1 through routine immunization services. The six countries with the highest numbers of infants who had not received MCV1 were Nigeria (3.3 million), Ethiopia (1.5 million), Democratic Republic of the Congo (DRC) (1.4 million), Pakistan (1.4 million), India (1.2 million), and Philippines (0.7 million), accounting for nearly half (48%) of the world’s total.

Estimated global MCV2 coverage nearly quadrupled from 18% in 2000 to 71% in 2019, largely because of an 86% increase in the number of countries providing MCV2, from 95 (50%) countries in 2000 to 177 (91%) in 2019 (Table 1). Six countries (Cameroon, Ethiopia, Liberia, Mali, Republic of the Congo, and Togo) introduced MCV2 in 2019.

Approximately 204 million persons received MCV during supplementary immunization activities (SIAs)^††^ in 55 countries in 2019; in addition, 9 million persons received MCV during measles outbreak response activities.

## Reported Measles Incidence

In 2019, all 194 countries conducted measles surveillance, and 193^§§^ (99%) had access to standardized quality-controlled laboratory testing through the WHO Global Measles and Rubella Laboratory Network. In spite of this, however, surveillance remains weak in many countries, and only 81 (52%) of 157 countries that reported discarded^¶¶^ cases achieved the sensitivity indicator target of two or more discarded measles and rubella cases per 100,000 population.

Countries report the number of incident measles cases*** to WHO and UNICEF annually using the Joint Reporting Form.^†††^ During 2000–2016, the number of reported measles cases decreased 84%, from 853,479 in 2000 to 132,490 in 2016. From 2000 to 2016, annual measles incidence decreased 88%, from 145 cases per 1 million (2000) to 18 (2016), the lowest reported incidence during this period; incidence then increased 567% to 120 per million in 2019, the highest since 2001 (Table 1). The percentage of reporting countries with annual measles incidence of <5 cases per 1 million population increased from 38% (64 of 169) in 2000 to 70% (125 of 179) in 2016, but then decreased to 46% (85 of 184) in 2019.

The number of measles cases increased 556% from 132,490 in 2016 to 869,770 in 2019, the most reported cases since 1996. Since 2016, the number of reported measles cases increased 1,606% in WHO’s African Region (AFR), 19,739% in the Region of the Americas (AMR), 194% in the Eastern Mediterranean Region (EMR), 2,282% in the European Region (EUR), 6% in the South-East Asia Region (SEAR), and 36% in the Western Pacific Region (WPR). In 2019, nine (5%) of 184 reporting countries (Central African Republic, DRC, Georgia, Kazakhstan, Madagascar, North Macedonia, Samoa, Tonga, and Ukraine) experienced large outbreaks, and in each of these countries, reported measles incidence exceeded 500 per 1 million population; these nine countries accounted for 631,847 (73%) of all reported cases worldwide during 2019.

Genotypes of viruses isolated from persons with measles were reported by 88 (62%) of 141 countries reporting at least one measles case in 2019. From 2005 to 2019, 20 of 24 recognized measles genotypes were eliminated by immunization activities. The number of genotypes detected decreased from 11 during 2005–2008, to eight during 2009–2014, six in 2016, five in 2017, and four during 2018–2019 (*3*). In 2019, among 8,728 reported sequences, 1,920 (22%) were genotype B3; six (0.1%) were D4; 6,774 (78%) were D8; and 28 (0.3%) were H1.^§§§^

## Measles Case and Mortality Estimates

A previously described model for estimating measles cases and deaths (*4*) was updated with annual vaccination coverage data, case data, and United Nations population estimates for all countries during 2000–2019, enabling derivation of a new series of disease and mortality estimates. For countries with anomalous estimates (e.g., a decrease in reported cases, but an increase in estimated deaths, or vice versa), the model was modified slightly to generate mortality estimates consistent with observed cases. Based on updated annual data, the estimated number of measles cases decreased 65%, from 28,340,700 in 2000 to 9,828,400 in 2019. During this period, estimated annual measles deaths decreased 62%, from 539,000 to 207,500 (Table 2). During 2000–2019, compared with no measles vaccination, measles vaccination prevented an estimated 25.5 million deaths globally (Figure).

**TABLE 2:** Estimated number of measles cases and deaths,* by World Health Organization (WHO) region — worldwide, 2000 and 2019

WHO region/Year (no. of countries in region)	Estimated no. of measles cases (95% CI)	Estimated no. of measles deaths (95% CI)	Estimated % measles mortality reduction from 2000 to 2019	Cumulative no. of measles deaths averted by vaccination, 2000–2019
**African**				
2000 (46)	10,727,500 (7,417,700–17,448,900)	346,400 (227,600–569,000)	57	13,620,000
2019 (47)	4,548,000 (3,266,700–8,376,100)	147,900 (99,500–271,100)		
**Americas**				
2000 (35)	8,800 (4,400–35,000)	NA^†^	NA	102,500
2019 (35)	102,700 (51,400–411,000)	NA^†^		
**Eastern Mediterranean**				
2000 (21)	2,565,800 (1,534,500–4,774,400)	40,000 (22,200–69,200)	33	2,877,900
2019 (21)	1,384,500 (717,900–3,201,000)	27,000 (14,700–49,500)		
**European**				
2000 (52)	816,600 (216,900–5,116,000)	350 (100–1,900)	66	101,300
2019 (53)	494,600 (192,800–6,571,400)	120 (20–1,700)		
**South-East Asia**				
2000 (10)	11,379,100 (8,937,200–15,299,200)	141,400 (102,000–194,600)	80	7,387,800
2019 (11)	2,655,000 (902,200–6,886,500)	28,700 (8,400–75,400)		
**Western Pacific**				
2000 (27)	2,843,000 (1,934,700–22,297,700)	10,900 (5,200–77,300)	65	1,385,500
2019 (27)	643,700 (127,600–18,007,600)	3,800 (500–75,100)		
**Totals**				
**2000 (191)**	**28,340,700 (20,045,300–64,971,300)**	**539,000 (357,200–911,900)**	**62**	**25,475,000**
**2019 (194)**	**9,828,400 (5,258,500–43,453,500)**	**207,500 (123,100–472,900)**		

**Abbreviations:** CI = confidence interval; NA = not applicable; UNICEF = United Nations Children’s Fund.

* The measles mortality model used to generate estimated measles cases and deaths is rerun each year using the new and revised annual WHO/UNICEF estimates of national immunization coverage (WUENIC) data, as well as updated surveillance data; therefore, the estimated number of cases and mortality estimates in this report might differ slightly from those in previous reports.

^†^ Estimated measles mortality was too low to allow reliable measurement of mortality reduction.

## Regional Verification of Measles Elimination

By the end of 2019, no WHO region had achieved and maintained measles elimination; 83 (43%) individual countries had been verified by independent regional commissions as having achieved or maintained measles elimination. The two countries verified in 2019 to have achieved elimination were Iran and Sri Lanka. No AFR country has yet been verified as having eliminated measles. The AMR had achieved verification of measles elimination in 2016; however, endemic measles transmission was reestablished in Venezuela in 2018 and in Brazil in 2019.

## Discussion

Despite substantial decreasing global measles incidence and measles-associated mortality during 2000–2016, the global measles resurgence that commenced during 2017–2018 continued in 2019 and marked a significant step backward in progress toward global measles elimination. Compared with the historic low in reported cases in 2016, reported measles cases increased 556% in 2019, with increases in numbers of reported cases and incidence in all WHO regions. Estimated global measles mortality increased nearly 50% since 2016. In all WHO regions, the fundamental cause of the resurgence was a failure to vaccinate, both in recent and past years, causing immunity gaps in both younger and some older age groups. Lessons can be learned from outbreaks in various countries, as well as from notable successes in countries such as China, Colombia, and India (*5*–*7*). Identifying and addressing gaps in population immunity will require additional strategies as outlined in the Immunization Agenda 2030^¶¶¶^ and the Measles-Rubella Strategic Framework 2021–2030 (*8*).

In 2019, the global increase in cases was driven by large outbreaks in several countries. Huge outbreaks occurred in DRC and Madagascar during 2018–2019 as a consequence of accumulations of large numbers of measles-susceptible children, which resulted from longstanding extremely low MCV1 coverage, no introduction of MCV2 into the immunization program, and suboptimal SIA implementation. Samoa’s outbreak resulted from a steady decline in MCV1 and MCV2 coverage during 2014–2018, exacerbated by a decline in vaccine confidence after two infant deaths occurred from an error in measles-mumps-rubella vaccine administration (*9*). Ukraine’s outbreak was the result of low vaccine confidence among health care professionals, low demand from the public, and challenges with vaccine supply, storage, and handling.**** Brazil’s outbreak was caused by previously unidentified immunity gaps, revealed by sustained transmission following multiple measles virus importations from the outbreak in neighboring Venezuela.^††††^

Outbreaks must be investigated to understand whether and why communities were missed by vaccination, so that immunization services can be strengthened to close population immunity gaps. Where low vaccination coverage exists in specific populations, assessment of behavioral and social drivers of low coverage is needed to inform the design and implementation of targeted strategies, whether related to practical factors such as limited access to services, or to social influences that affect confidence and motivation to receive vaccination. Programs need to work to achieve and sustain the trust of parents and communities to ensure understanding that receipt of vaccination is in their children’s best interests. Programs should always be well prepared to respond to any vaccine-related adverse event in a timely and effective manner to obviate fears and hesitancy that can erode progress.

The findings in this report are subject to at least three limitations. First, large differences between estimated and reported incidence indicate overall low surveillance sensitivity, making comparisons between regions difficult to interpret. Second, some countries have multiple measles surveillance systems and choose which data to submit to WHO. In 2019, for example, Chad reported 1,882 cases to WHO from one surveillance system, but another surveillance system identified 26,623 suspected measles cases. Finally, the measles mortality model estimates might be biased upward or downward by inaccurate model inputs, including vaccination coverage and surveillance data.

In 2020, the coronavirus disease 2019 pandemic has produced increased programmatic challenges, leading to fewer children receiving vaccinations and poorer surveillance (*10*). Progress toward measles elimination during and after the pandemic will require strategies to integrate catch-up vaccination policies into essential immunization services, assurance of safe provision of services, engagement with communities to regain trust and confidence in the health system, and rapid outbreak response.

As outlined in the Immunization Agenda 2030, a global immunization strategy for 2021–2030, further progress toward achieving measles elimination goals will require strengthening essential immunization systems to increase 2-dose coverage, identify and close historical immunity gaps through catch-up vaccination to prevent outbreaks, improve surveillance and preparedness for rapidly responding to outbreaks, and leverage measles as a tracer and guide to improving immunization programs (*8*).

**Summary**

**What is already known about this topic?**

All six World Health Organization (WHO) regions have a measles elimination goal.

**What is added by this report?**

During 2000–2016, annual reported measles incidence decreased globally; however, measles incidence increased in all regions during 2017–2019. Since 2000, estimated measles deaths decreased 62% and measles vaccination has prevented an estimated 25.5 million deaths worldwide. No WHO region has achieved and maintained measles elimination.

**What are the implications for public health practice?**

To achieve regional measles elimination goals, additional strategies are needed to help countries strengthen routine immunization systems, identify and close immunity gaps, and improve case-based surveillance.

**FIGURE:**
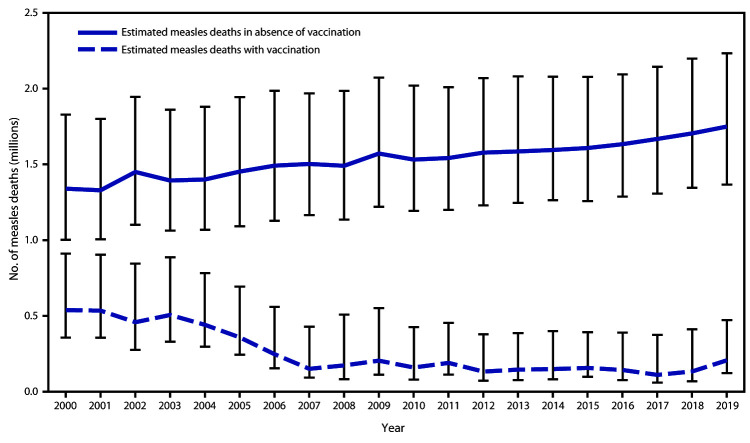
Estimated number of annual measles deaths with vaccination and in the absence of vaccination — worldwide, 2000–2019* * Deaths prevented by vaccination are estimated by the area between estimated deaths with vaccination and those without vaccination (cumulative total of 25.5 million deaths prevented during 2000–2019). Vertical bars represent upper and lower 95% confidence intervals around the point estimate.

## Footnotes

* The coverage milestone is to be achieved by every country, whereas the incidence and mortality reduction milestones are to be achieved globally.

† The Global Vaccine Action Plan is the implementation plan of the Decade of Vaccines, a collaboration between WHO; UNICEF; the Bill and Melinda Gates Foundation; the National Institute of Allergy and Infectious Diseases; the African Leaders Malaria Alliance; Gavi, the Vaccine Alliance; and others to extend the full benefit of immunization to all persons by 2020 and beyond. In addition to 2015 targets, it also set a target for measles and rubella elimination in five of the six WHO regions by 2020. https://www.who.int/immunization/global_vaccine_action_plan/en.

§ Measles elimination is defined as the absence of endemic measles virus transmission in a region or other defined geographic area for ≥12 months, in the presence of a high-quality surveillance system that meets targets of key performance indicators.

¶ Calculated for MCV1, among children aged 1 year or, if MCV1 is given at age ≥1 year, among children aged 24 months. Calculated for MCV2 among children at the recommended age for administration of MCV2, per the national immunization schedule. WHO/UNICEF estimates of national immunization coverage are available at https://www.who.int/immunization/monitoring_surveillance/data/en.

** In 2000, 191 countries were requested to report to WHO; by 2019, 194 member states were requested to report because of the creation of new countries. For district level coverage, only countries that reported data are in the numerator, while the denominator is all WHO countries in that year (191–194) regardless of whether they reported data.

†† SIAs generally are carried out using two target age ranges. An initial, nationwide catch-up SIA focuses on all children aged 9 months–14 years, with the goal of eliminating susceptibility to measles in the general population. Periodic follow-up SIAs then focus on all children born since the last SIA. Follow-up SIAs generally are conducted nationwide every 2–4 years and focus on children aged 9–59 months; their goal is to eliminate any measles susceptibility that has developed in recent birth cohorts because of low MCV coverage and to protect children who did not respond to MCV1. Data on SIAs by country are available at https://www.who.int/immunization/monitoring_surveillance/data/Summary_Measles_SIAs.xls?ua.

§§ Sao Tome and Principe did not have access to standardized quality-controlled testing by the WHO Measles and Rubella Laboratory Network in 2019.

¶¶ A discarded case is defined as a suspected case that has been investigated and determined to neither be measles nor rubella using 1) laboratory testing in a proficient laboratory or 2) epidemiologic linkage to a laboratory-confirmed outbreak of a communicable disease that is not measles or rubella. The discarded case rate is used to measure the sensitivity of measles surveillance.

*** https://apps.who.int/immunization_monitoring/globalsummary/timeseries/tsincidencemeasles.html; data as of July 15, 2020. Only countries that reported data are in the numerator and denominator.

††† https://www.who.int/immunization/monitoring_surveillance/routine/reporting/en/ Twenty-five countries did not report case data in 2000: Algeria, Austria, Belgium, Comoros, Equatorial Guinea, Fiji, Finland, Germany, Guinea-Bissau, Ireland, Libya, Mauritania, Monaco, Montenegro, North Korea, Samoa, Saudi Arabia, Seychelles, Slovenia. Solomon Islands, South Sudan, Switzerland, Timor-Leste, Tuvalu, and Yemen. Fifteen countries did not report case data in 2016: Belgium, Cabo Verde, Cook Islands, Haiti, Italy, Kiribati, Marshall Islands, Monaco, Morocco, Mozambique, Niue, Samoa, Singapore, Tuvalu, and Vanuatu. Ten countries did not report case data in 2019: Belgium, Djibouti, Malta, Marshall Islands, Morocco, North Korea, Palau, Solomon Islands, Switzerland, and the United States. Countries do not provide WHO with their reasons for not reporting case data.

§§§ http://www.who-measles.org/; data as of September 5, 2020.

¶¶¶ Immunization Agenda 2030 is the global vision and strategy to extend the benefits of vaccines to everyone, everywhere, developed by immunization stakeholders and endorsed by the World Health Assembly in 2020. https://www.who.int/immunization/immunization_agenda_2030/en/.

**** Strategic Response Plan for the Measles Emergency in the WHO European Region, September 2019–December 2020. Copenhagen, Denmark, 2019. https://www.euro.who.int/_data/assets/pdf_file/0020/414182/WHO-Measles-Emergency-v8a_hires_pages.pdf.

†††† https://www.paho.org/en/documents/epidemiological-update-measles-28-february-2020.
